# Synthesis and Cytotoxic Activity of New 1,3,4-Thiadiazole Thioglycosides and 1,2,3-Triazolyl-1,3,4-Thiadiazole *N*-glycosides

**DOI:** 10.3390/molecules24203738

**Published:** 2019-10-16

**Authors:** Fahad M. Alminderej, Hussein H. Elganzory, Mohamed N. El-Bayaa, Hanem M. Awad, Wael A. El-Sayed

**Affiliations:** 1 Chemistry Department, College of Science, Qassim University, Buraidah 51452, Saudi Arabia; f.alminderej@qu.edu.sa; 2 Photochemistry Department, National Research Centre, Cairo 12311, Egypt; mnb6600@yahoo.com; 3 Tanning Materials and Leather Technology Department, National Research Centre, El-Behouth St, Dokki, Cairo 12311, Egypt; hanem_awad@yahoo.com

**Keywords:** click chemistry, 1,3,4-thiadiazoles, 1,2,3-triazoles, glycosides, cytotoxic, HCT-116, MCF-7

## Abstract

New 1,3,4-thiadiazole thioglycosides linked to substituted arylidine systems were synthesized via glycosylation of the prepared 1,3,4-thiadiazole thiol compounds. Click strategy was also used for the synthesis of new 1,3,4-thiadiazole and 1,2,3-triazole hybrid glycosides by reaction of the acetylenic derivatives with different glycosyl azids followed by deacetylation process. The cytotoxic activities of the prepared compounds were studied against HCT-116 (human colorectal carcinoma) and MCF-7 (human breast adenocarcinoma) cell lines using the MTT assay. The results showed that the key thiadiazolethione compounds **2** and **3**, the triazole glycosides linked to *p*-methoxyarylidine derivatives **14** and **15** in addition to the free hydroxyl glycoside **20** were found potent in activity comparable to the reference drug doxorubicin against MCF-7 human cancer cells. The acetylenic derivative **2** and glycoside **20** were also found highly active against HCT-116 cell lines.

## 1. Introduction

**Figure 1:**
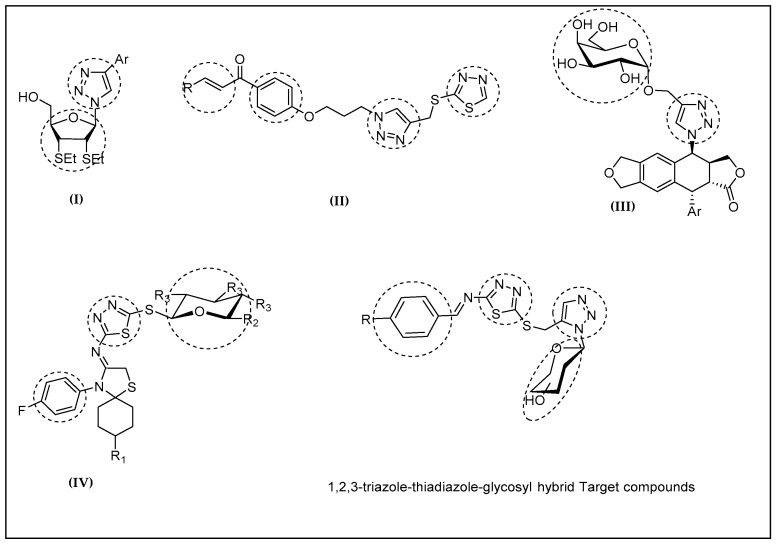
Anticancer 1,2,3-triazole glycosides (**I**, **II**, and **III**), 1,3,4-thiadiazole glycosides (**IV**), and targeted hybrid compounds.

The enhanced global encumbrance of cancer in the last decades devoted the extensive research to the discovery of novel selective potent candidates with promising ability either to destroy cancer cells or limit their proliferation. Click chemistry is now considered as one of the most efficient strategies providing rapid and mild routes for novel bioorganic compounds such as anticancer candidates. The strategy involves the Cu (I)-catalyzed 1,3-dipolar cycloaddition of an azide and alkyne leading, with outstanding selectivity, to the formation of functionalized 1,4-disubstitued 1,2,3-triazole under mild conditions with high yields [1]. The formed products via click cycloaddition possess the 1,2,3-triazole core which is well known to be incorporated in a large number of compounds reported by their important pharmacological properties [2,3]. Triazole-modified analogs possessing significant anticancer activity have been synthesized by means of an azide–alkyne click chemistry strategy [4]. In addition to its importance as a basic motif of numerous nucleosides analogs featured with antiviral, as anti-HIV, or cytostatic activities [5,6,7], the triazole nucleus was also revealed with potential anticancer activity [8,9,10]. The interesting structural characteristics involving the superb plain immutability to metabolic transformation, aromaticity nature, as well as the high dipole moment and H-bonding ability of the triazole core, prompted its potential usefulness as a connecting group [11,12]. Consequently, such ring motif could be a useful linker for joining two different kinds of functionalities with biological interest leading to the formation of useful hybrid molecules. These features allowed 1,2,3-triazoles and their incorporating compounds to be exploited for designing a variety of scaffolds with substantial medicinal characteristics such as anticancer and enzyme inhibition activities [13,14,15]. It has been revealed that the attachment of suitable aromatic system to the triazole core, compound **I** (Figure 1), is important and such conjunction resulted in a positive influence for enhancing bioactivity.

Thiadiazole ring system, being featured as a structural bio-isostere of pyrimidine and oxadiazole, has shown numerous biological activities including anticancer, antifungal, antiviral, antidiabetic, analgesic, antieplileptic, antibacterial, and anti-inflammatory activities [16,17,18,19,20]. The enhanced liposolubility imparted by the sulfur atom of the thiadiazole core in addition to the mesoionic nature allowed compounds incorporating such motif to interact with biological targets with distinct affinities after penetration across cellular membranes. Recent studies displayed the ability of 1,3,4-thiadiazole compounds to inhibit a variety of molecular targets, including kinases which allowed their activity against different cancer cell lines presenting them as a promising scaffold for antitumor drug discovery [16,17,18,19,20,21,22,23,24,25].

On the other hand, nucleoside analogues have been recognized as active drug candidates for the treatment of different cancer forms. A number of nucleoside analogues such as Cytarabine, Azacitidine, Capecitabine, Floxuridine, and Decitabine are indeed ratified with antitumor activity [26]. Figure 1 displays a number of reported highly active 1,3,4-thiadiazole and triazole glycosides (**I**, **III**, and **IV**) and the triazole–thiadiazole hybrid **II** against different cancer cells [7,8,9,27,28]. Such significances and our interest in synthesizing new active glycosyl heterocycles [29,30,31,32,33], enhanced our foresight that the 1,3,4-thiazoles and triazoles motif hybrid compounds and their sugar linked products could be useful systems for designing novel cytotoxic agents. In the current study, new thioglycosides of arylamino-1,3,4-thiadiazole and glycosyl-1,3,4-thiadiazole-triazole conjugates were synthesized and studied for their cytotoxic activity against human colorectal carcinoma and breast adenocarcinoma cell lines.

## 2. Results and Discussion

### 2.1. Chemistry

**Scheme 1:**
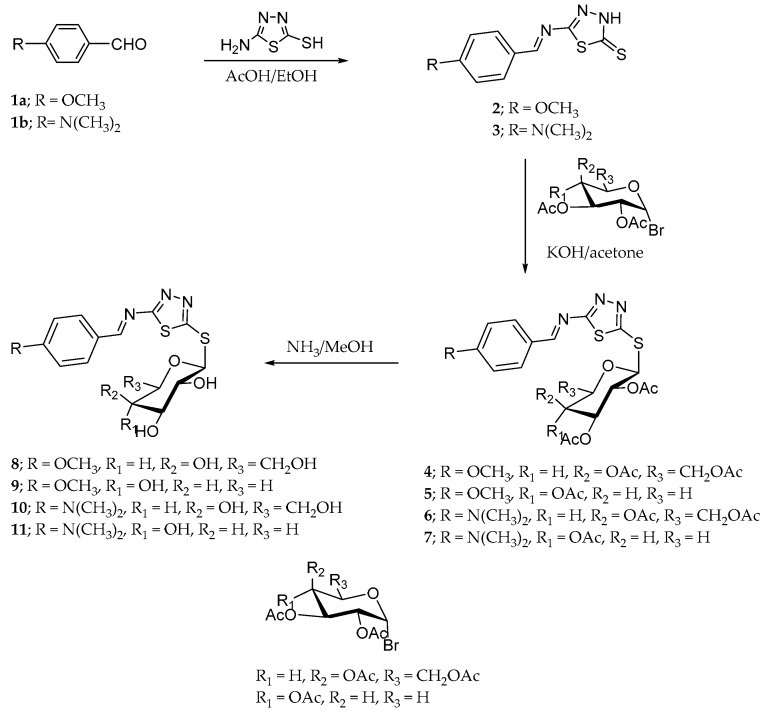
Synthesis of thiadiazole glycosides based arylidine system.

In the current study, two, structurally, related glycosyl heterocyclic systems incorporating different glycol-linkages were synthesized via synthetic strategies starting with available simple molecules. The functionalized 1,3,4-thiadiazole thione bases **2** and **3**, which are linked to arylidine systems, were obtained via reaction of aromatic aldehydes; namely *p*-methoxybenzaldehyde or *p*-*N*-dimethylaminobenazaldehyde with 3-amino-1,3,4-thiadiazole-5-thiol in acidic ethanolic solution. The obtained products were reacted with the acetylated bromosugar, tetraacetyl-d-galactopyranosyl- or triacetyl-d-xylopyranosyl bromide to afford the corresponding thioglycosides **4**–**7**, respectively. The particular ^1^H-NMR spectra revealed the signal attributed to anomeric hydrogen of the glycosyl unit as doublet at δ 5.20–5.55 ppm, exhibiting coupling constants in the range 9.4–9.8 Hz indicating the β-type orientation of the thioglycosidic attachment. For the compounds possessing β-*N*-glycosidic part near to the C=S group, the anomeric proton has been reported to be exist [34,35,36] at higher chemical shifts (δ 6.5–7.2 ppm). Such relatively raised δ values in such type of structures are explained by the anisotropic deshielding influence of the C=S. The thio-linkage and β-conformation in the thioglycosides **4**–**7** were also confirmed by the assigned signal at *δ* 85.1–86.5 ppm for the anomeric C-1 in the ^13^C-NMR spectra in addition to the absence of the well-characteristic signal corresponding to the C=S group which accounts for the attachment of the glycosyl part at the sulfur atom and the regioselectivity of the glycosylation reaction. The derived thioglycosides with unprotected OH groups **8**–**11** were obtained by deacetylation of the acetylated **4**–**7**, respectively, by the treatment with methanolic ammonia solution (Scheme 1). The IR spectra of the deacetylated thio-glycoside **8**–**11** showed the absorption bands which were attributed to the hydroxyl groups in the sugar unit and disappearance of the characteristic C=O of the acetyl bands which was also confirmed by the corresponding ^1^H-NMR spectra which did not possess the methyl proton signals and instead displayed the hydroxyl peaks.

**Scheme 2:**
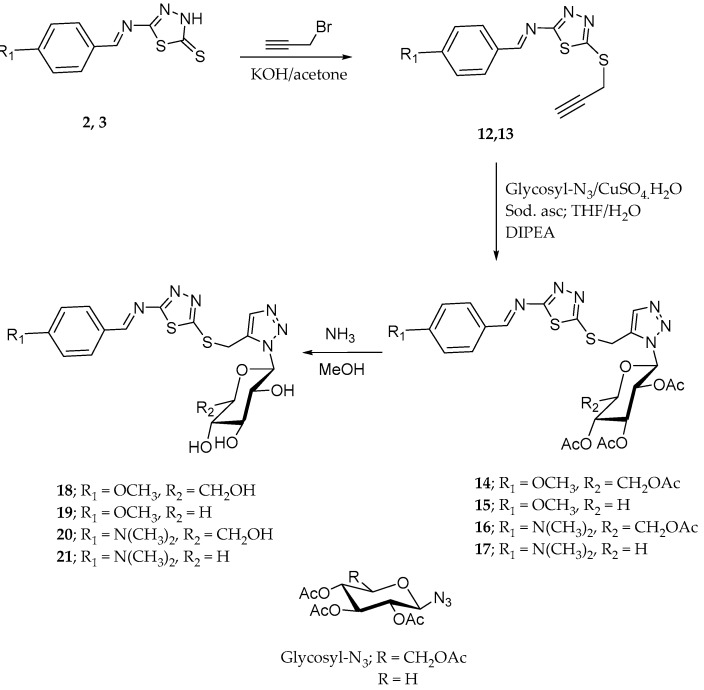
Synthesis of thiadiazolyl-1,2,3-triazole glycosides.

In order to add a 1,2,3-trizole core as an additional ring system to the previous arylidine thiadiazole compounds, click 1,3-dipolar cycloaddition strategy was applied for synthesizing the target compounds. The necessary terminal acetylenic active center was formed by reaction of the thioles **2** and **3** with propargyl bromide in a basic medium. The produced terminal acetylenic compounds **12** and **13** were reacted with glycopyranosyl azides namely; 2,3,4,6-tetra-*O*-acetyl-d-gluco- or xylopyranosyl azides by means of Cu(I) catalysis in 1,3-dipolar cycloaddition reactions by applying click conditions, to give the targeted 1,2,3-triazole-*N*-glycoides, in 73–76% yields. In the latter reactions, sodium ascorbate and copper sulfate pentahydrate coupled system was utilized for generation of necessary Cu(I) species by the in-situ reduction of copper (II) salt. THF/water (2:1) mixed solvent system was found to be the most efficient solvent system resulting, regioselectively, in highest yields of the desired click products **14**–**17**. The ^1^H-NMR spectra of the *N^1^*-glycosyl-1,2,3-triazolyl linked to thiadiazole system showed the corresponding signals assigned for the glycopyranosyl protons including the protons of the CH_3_C=O and signals of the remaining protons in the sugar part. The revealed *J* values corresponding to the anomeric hydrogen (H-1) at 9.6–10.2 Hz also confirmed the β-*N*-glycosidic linkage mode of the glycopyranosyl ring to the triazole motif. The afforded acetylated 1,2,3-triazole glycosides **14**–**17** were deacetylated by using a saturated ammonia/methanol solution which led to the formation of the derived 1,2,3-triazole-*N*-glycosides **18**–**21**, respectively (Scheme 2). The absence of the carbonyl band of CH_3_C=O in the assigned infra-red spectra and appearance of the particular absorptions corresponding to –OH groups evidenced the deacetylation process. Furthermore, their ^1^H-NMR spectra revealed the signals assigned to the hydroxyl protons and, in addition, disappearance of the signals of the acetyl groups of the protected precursors which is in complete stratification with the structures of the resulted free hydroxy glycosides.

### 2.2. Cytotoxic Activity

**Figure 2:**
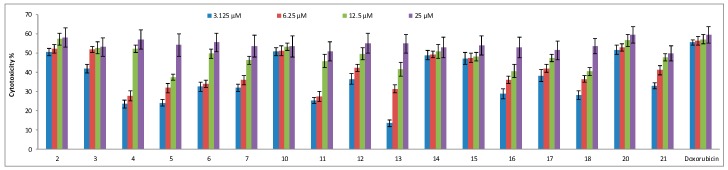
Dose dependent cytotoxic activities of the compounds against HCT-116 cancer cells according to the MTT assay.

**Figure 3:**
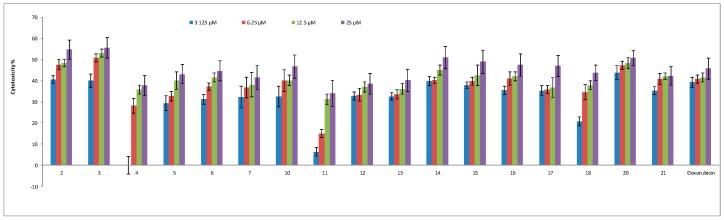
Dose dependent cytotoxic activities of the compounds against MCF-7 cancer cells according to the MTT assay.

The synthesized compounds were examined in vitro for their activities on HCT-116 (human colorectal carcinoma) and MCF-7 (human breast adenocarcinoma) cell lines using the MTT assay. The percentages of intact cells were calculated and compared to those of the control. Activities of tested compounds against the two cell lines were compared to the activity of doxorubicin as well. All compounds suppressed the two human cells in an insignificant dose-dependent manner as shown in Figure 2 and Figure 3. In order to study the efficacy of the synthesized compounds, a comparison of the cytotoxic effect of each compound has been related to the cytotoxicity of the reference drug as follow. In case of HCT-116 human colorectal carcinoma cells, Figure 2, Table 1, shows that compounds **2** and **20** had equipotent activities. Furthermore, the tested compounds **10**, **14**, **3**, **15**, and **12**, respectively, had slightly lower cytotoxic activities than that of doxorubicin. The rest of the tested compounds had significantly less cytotoxic activities when compared to that of the reference drug.

The results of cytotoxic activities investigation against MCF-7 human breast cancer cells showed that compounds **3**, **2**, **20**, and **14**, respectively, had significantly more potent cytotoxic activities. Furthermore, compounds **15**, **16**, **21**, and **10** had comparable activities to that of doxorubicin, whereas compounds **17**, **6**, **18**, **7**, **5**, **13**, and **12** had insignificantly less cytotoxic activities than the latter derivatives. The two other compounds, **4** and **11**, in the current investigation were significantly less active compared to that of doxorubicin Figure 3, Table 1.

From the above results, it can be revealed that compounds **2** and **20** are nonspecifically active candidate drugs on both colon and breast human cancer types. It has also been shown that although compounds **3**, **14**, and **15** are active against both two cancer cell lines, they showed more specificity against human breast cancer lines. On the other hand, compounds **16** and **21** are specifically active candidate drugs against human breast cancer but not active on human colon cancer types.

In correlation of the afforded activity results with the structural characteristics of the highly active compounds, it was revealed that two candidates comprising two types of synthesized compounds, glycosyl-1,2,3-triazoles and 1,3,4-thiadiazole-thiol, were found to be of high activities against both cell lines. The results generally showed the importance of the 1,2,3-triazole ring system, introduced by click reactions, in highly active triazole glycosides **14**–**16**, **20**, and **21,** in compounds with *N*-dimethylaminoarylidine part against either MCF-7 or HCT-116 cancer cells. It was well observed that the activity was lost in structurally related analogs, which lacks such ring system in compounds **4**–**6** and **11**. It is noteworthy to mention that the obtained inhibition results also indicated that the attachment of the glucopyranosyl part with free unprotected hydroxyl groups to the 1,2,3-triazole ring system afforded *N*-glycosides with high cytotoxic activity more than its xylopyranosyl analog and also more than its acetylated precursors (the triazole glycosides **21** and **16**). However, this was not the case when the phenyl ring in the arylidine part was substituted with *p*-methoxy substituent since the protected *N*-glycosides were found to be more active against both cells than their derived deacetylated compounds. 

The 1,3,4-thiadiazole glycosides incorporating thio-linkage attachment of the thiadiazole ring to the glycosyl part did not show high activities. The latter finding is, indeed, much more useful to show the importance of the free thiol-thione group at *C*-5 in the thiadiazole ring since both thiadiazolethione compounds **2** and **3** were highly active and such activities were lost in their derived glycosides in which such group was blocked. This conclusion was also supported by the afforded activity results of the acetylenic compounds **12** and **13,** which showed much lower cytotoxic activities than their starting free thiol compound. On the other hand, the activity of the deprotected glycoside **10** against HCT-116 cell was improved compared to that of the glycoside **6**, but the activity of the deprotected **11** was significantly declined compared to that of **7**, especially against MCF-7 cancer cell which may refer to the influence of nature of sugar unit. The acetylenic derivative **12** with the *para*-methoxy substituent in the phenyl ring showed better activity, against HCT-116 cells, than its *para*-*N*-dimethylamino analog **13** which was also observed for the thiadiazole compounds **2** and **3**.

## 3. Experimental

### 3.1. Synthesis

#### General Procedures

Melting points were determined on a Böetius PHMK apparatus (Veb Analytik, Dresden, Germany). TLC was performed using aluminum plates, pre-coated with silica gel 60 or 60 F254 (Merck, Munchen, Germany.) and visualized by iodine or UV light (254 nm, Merck, Darmstadt, Germany). The NMR spectra were recorded on a Varian Gemini 300 and Bruker DRX 400 spectrometer (Varian, Palo Alto, CA, USA) at 25 °C. ^1^H- and ^13^C-NMR signals were referenced to TMS and the solvent shift ((CD_3_)_2_SO δ H 2.50 and δ C 39.5). Coupling constants are given in Hz and without sign. The IR-spectra were recorded (KBr) on a Jasco FT/IR-410 instrument (Jasco, Easton, PA, USA). Microanalyses were operated using Perkin Elmer 240 instrument (Perkin Elmer 240, Waltham, MA, USA) and satisfactory results within the accepted range (±0.40) of the calculated values were obtained. All reagents and solvents were of commercial grade. The cytotoxic activity of the synthesized compounds was studied at National Research Center, Cairo, Egypt. The glycosyl azides were prepared as previously reported [37,38].

### 3.2. Synthesis of 5-[(4-arylidine)amino]-[1,3,4]thiadiazole-2-thiol (***2***,***3***)

To a solution of 4-methoxybenzaldehyde or 4-*N*,*N*-dimethylaminobenzaldehyde (10 mmol) in ethanol (30 mL) was added a catalytic amount of glacial acetic acid (0.5 mL) followed by addition of 3-amino-1,3,4-thiadiazole-5-thiol (10 mmol). The reaction mixture was heated at reflux temperature for 7 h, then allowed to stand overnight at room temperature. The precipitated solid was filtered, washed with ethanol, and recrystallized from ethanol to afford compounds **2** or **3**, respectively.

#### 3.2.1. 5-((4-Methoxybenzylidene)amino)-1,3,4-thiadiazole-2(3*H*)-thione (**2**)

Yield: 79%; m.p. 210–211 °C; IR (KBr) cm^−1^, ν: 3285 (NH), 3065 (C-H), 1605 (C=N); ^1^H-NMR (DMSO-d_6_) δ/ppm: 3.85 (s, 3H, OCH_3_), 7.24 (d, 2H, *J* = 8.8 Hz, Ar-H), 7.84 (d, 2H, *J* = 8.8 Hz, Ar-H), 8.61 (s, 1H, N=CH), 14.40 (s, 1H, NH). ^13^C-NMR spectrum, δ, ppm: 53.2 (CH_3_), 112.5–159.9 (6Ar-C, C=N, thiadiazole-C), 164.9 (thiadiazole-C), 182.2 (C=S). Analysis calcd. for C_10_H_9_N_3_OS_2_ (251.33): C, 47.79; H, 3.61; N, 16.72. Found: C, 47.55; H, 3.50; N, 16.65%.

#### 3.2.2. 5-((4-(Dimethylamino)benzylidene)amino)-1,3,4-thiadiazole-2(3*H*)-thione (**3**)

Yield: 77%; m.p. 2014–2016 °C; IR (KBr) cm^−1^, ν: 3290 (NH), 3055 (C-H), 1608 (C=N); ^1^H-NMR (DMSO-d_6_) δ/ppm: 2.06,3.08 (2s, 6H, N(CH_3_)_2_), 6.68 (d, 2H, *J* = 8.8 Hz, Ar-H), 7.24 (s, 1H, N=CH), 7.81 (d, 2H, *J* = 8.8 Hz, Ar-H), 14.40 (s, 1H, NH). Analysis calcd. for C_11_H_12_N_4_S_2_ (264.37): C, 49.98; H, 4.58; N, 21.19. Found: C, 49.74; H, 4.66; N, 21.02%.

### 3.3. Synthesis of 4-(((5-((O-acetylglycopyranosyl)thio)-1,3,4-thiadiazol-2-yl)imino)methyl)-4-substituted aniline (***4***–***7***)

Potassium hydroxide (12 mmol) suspended in water (1 mL) was added to a well-stirred solution of the (arylidine)amino]-[1,3,4]thiadiazole derivative **2** or **3** (10 mmol) in acetone (15 mL). The galactosyl or xylxosyl bromide (12 mmol) dissolved in acetone (10 mL) was added and the reaction mixture was stirred at r.t. for 18 h (TLC: Pet. ether/ethyl acetate; 4:1). The solvent was evaporated and the residue was treated with pet. ether (40–60, 2 × 15 mL) to form a solid which was filtered and dried to give compounds **4**–**7**.

#### 3.3.1. (5-(2,3,4,6-Tetra-O-acetyl-β-d-galactopyranosyl)thio-[1,3,4]thiadiazol-2-yl)-(4- methoxy-benzylidene)amine (**4**)

Yield: 73%; m.p. 112–113 °C; IR (KBr) cm^−1^, ν: 3072 (C-H), 1752 (C=O), 1610 (C=N); ^1^H-NMR (DMSO-d_6_) δ/ppm: 1.84, 1.99, 2.08, 2.52 (4s, 12H, CH_3_CO), 3.88 (s, 3H, OCH_3_), 4.18–4.22 (dd, 1H, *J* = 3.2, *J* = 11.2 Hz, H-6*’*), 4.25–4.28 (dd, 1H, *J* = 11.2, *J* = 3.8 Hz, H-6′′), 4.31–4.32 (m, 1H, *J* = 4.7 Hz, H-5′), 4.66–4.68 (m, 1H, H-4′), 5.20 (d, 1H, *J* = 9.8, H-1′), 5.23–5.26 (dd, 1H, *J* = 9.8, *J* = 8.8 Hz, H-2′), 5.27 (t, 1H, *J* = 8.8 Hz, H-3′), 6.90–6.97 (d, 2H, *J* = 8.6 Hz, Ar-H), 8.23 (d, 2H, *J* = 8.6 Hz, Ar-H), 8.54 (s, 1H, N=CH). ^13^C-NMR (DMSO-d_6_) δ/ppm: 20.5, 20.8, 20.8, 20.9 (4CH_3_), 56.14 (OCH_3_), 68.36 (C-6), 70.64 (C-4), 72.33 (C-2), 75.6 (C-3), 75.9 (C-5), 85.1 (C-1), 114.97–143.61 (Ar-C), 164.1, 164.26 (C=N, thiadiazole-C), 168.5, 168.7, 168.90, 169.1, 170.1 (4C=O, thiadiazole-C). Analysis calcd. for C_24_H_27_N_3_O_10_S_2_ (581.62): C, 49.56; H, 4.68; N, 7.22. Found: C, 49.39; H, 4.60; N, 7.37%.

#### 3.3.2. (5-(2,3,4-Tri-O-acetyl-β-d-xylopyranosyl)thio-[1,3,4]thiadiazol-2-yl)-(4-methoxy-benzylidene)amine (**5**)

Yield: 75%; yellowish foam; IR (KBr) cm^−1^, ν: 3070 (C-H), 1750 (C=O), 1612 (C=N); ^1^H-NMR (DMSO-d_6_) δ/ppm: 1.64, 1.79, 2.00 (3s, 9H, CH_3_CO), 3.25–3.33 (m, 1H, H-5′′), 3.43–5.46 (m, 1H, H-5′), 3.76 (s, 3H, OCH_3_), 3.99–4.15 (m, 1H, H-4′), 4.78–4.82 (m, 1H, H-2′), 5.38 (d, 1H, *J* = 9.4 Hz, H-1′), 5.88 (t, 1H, *J* = 8.6 Hz, H-3′), 7.04 (d, 2H, *J* = 7.4 Hz, Ar-H), 7.86 (d, 2H, *J* = 7.1 Hz, Ar-H), 8.46 (s, 1H, N=CH). Analysis calcd. for C_21_H_23_N_3_O_8_S_2_ (509.55): C, 49.50; H, 4.55; N, 8.25. Found: C, 49.74; H, 4.41; N, 8.38%.

#### 3.3.3. (4-Dimethylaminobenzylidene)-(5-(2,3,4,6-tetra-*O*-acetyl-β-d-galactopyranosyl) thio-[1,3,4]thiadiazol-2-yl)-amine (**6**)

Yield: 72%; m.p. 139–140 °C; IR (KBr) cm^−1^, ν: 3048 (C-H), 1748 (C=O), 1615 (C=N); ^1^H-NMR (DMSO-d_6_) δ/ppm: 2.01, 2.08, 2.12, 2.18 (4s, 12H, CH_3_CO), 2.99, 3.14 (2s, 6H, N(CH_3_)_2_), 3.56–3.62 (dd, 1H, *J* = 2.8, *J* = 10.8 Hz, H-6′′), 3.91–4.01 (m, 1H, H-6*’*), 4.12–4.21 (m, 1H, H-5′), 4.44–4.47 (dd, 1H, *J* = 7.9, *J* = 8.8 Hz, H-4′), 5.01–5.04 (dd, 1H, *J* = 8.6, *J* = 9.8 Hz, H-2′), 5.22 (d, 1H, *J* = 9.8 Hz, H-1′), 5.51 (t, 1H, *J* = 8.6 Hz, H-3′), 7.76 (d, 2H, *J* = 8.8 Hz, Ar-H), 7.84 (d, 2H, *J* = 8.8 Hz, Ar-H), 9.76 (s, 1H, N=C*H*). ^13^C-NMR (DMSO-d_6_) δ/ppm: 20.2, 204, 20.6, 20.8 (4CH_3_), 40.1 (2CH_3_), 61.8 (C-6), 67.6 (C-4), 72.9 (C-2), 75.2 (C-3), 77.2 (C-5), 86.5 (C-1), 111.0–154.4 (Ar-C, thiadiazole-C), 166.5 (C=N), 167.9, 168.3, 168.5, 169.3, 170.1 (4C=O, thiadiazole-C). Analysis calcd. for C_25_H_30_N_4_O_9_S_2_ (594.66): C, 50.50; H, 5.09; N, 9.42. Found: C, 50.19; H, 5.02; N, 9.32%.

#### 3.3.4. (4-Dimethylamino-benzylidene)-(5-(2,3,4-tri-*O*-acetyl-β-d-xylopyranosyl)thio-[1,3,4]thiadiazol-2-yl)-amine (**7**)

Yield: 69%; yellowish foam; IR (KBr) cm^−1^, ν: 3064 (C-H), 1752 (C=O), 1615 (C=N); ^1^H-NMR (DMSO-d_6_) δ/ppm: 1.95, 2.01, 2.03 (3s, 9H, CH_3_CO), 2.88, 3.03 (2s, 6H, N(CH_3_)_2_), 3.34–3.44 (m, 1H, H-5′′), 4.76–4.78 (dd, 1H, *J* = 10.8, *J* = 3.4 Hz, H-5′), 4.89–4.91 (m, 1H, H-4′), 5.10–5.11 (m, 1H, H-2′), 5.55 (d, 1H, *J* = 9.6 Hz, H-1′), 5.63 (t, 1H, *J* = 8.2 Hz, H-3′), 6.62 (d, 2H, *J* = 8.9 Hz, Ar-H), 7.65 (d, 2H, *J* = 8.8 Hz, Ar-H), 9.65 (s, 1H, N=CH). Analysis calcd. for C_22_H_26_N_4_O_7_S_2_ (522.59): C, 50.56; H, 5.02; N, 10.72. Found: C, 50.36; H, 4.94; N, 10.83%.

### 3.4. Synthesis of 4-(((5-((glycopyranosyl)thio)-1,3,4-thiadiazol-2-yl)imino)methyl)-N,N-dimethylaniline (***8***–***11***)

The acetylated thioglycosides **4**–**7** (2 mmol) were dissolved in a saturated gaseous ammonia solution in dry methanol (20 mL) and the resulting solution was stirred at room temperature for 18 h. The solvent was evaporated under reduced pressure and the residue was with dissolved in ethanol with warming at 40 °C, then left to stand at room temperature for 2 h. The solvent was reduced under vacuum and the precipitate was filtered and dried to give the deacetylated thioglycosides **8**–**11**, respectively.

#### 3.4.1. (5-(β-d-Galactopyranosyl)sulfanyl-[1,3,4]thiadiazol-2-yl)-(4-methoxy-benzylidene)-amine (**8**)

Yield: 69%; m.p. 164–165 °C; IR (KBr) cm^−1^, ν: 3440–3425 (OH), 3055 (C-H), 1615 (C=N); ^1^H-NMR (DMSO-d_6_) δ/ppm: 3.53 (s, 3H, OCH_3_), 3.55–3.66 (m, 2H, H-6′,6′′), 3.73–3.75 (m, 1H, H-5′), 3.96–4.16 (m, 2H, H-4′,3′), 4.22–4.24 (m, 1H, OH), 4.52–4.73 (m, 2H, OH, H-2′), 5.91–5.10 (m, 2H, 2OH), 5.79 (d, 1H, *J* = 8.6 Hz, H-1′), 7.54 (d, 2H, *J* = 8.5 Hz, Ar-H), 8.30 (d, 2H, *J* = 8.5 Hz, Ar-H), 8.55 (s, 1H, N=C*H*). ^13^C-NMR spectrum, δ, ppm: 56.21 (OCH_3_), 60.6 (C-6′), 63.3 (C-4′), 68.2 (C-2′), 71.1 (C-3′), 75.7 (C-5′), 79.7 (C-1′), 111.5–159.9 (Ar-C), 166.7 (C=N), 174.7, 174.9 (thiadiazole-C-3,5). Analysis calcd. for C_16_H_19_N_3_O_6_S_2_ (413.47): C, 46.48; H, 4.63; N, 10.16. Found: C, 46.61; H, 4.55; N, 10.07%.

#### 3.4.2. (5-(β-d-Xylopyranosyl)sulfanyl-[1,3,4]thiadiazol-2-yl)-(4-methoxybenzylidene)-amine (**9**)

Yield: 67%; yellowish foam; IR (KBr) cm^−1^, ν: 3445–3420 (OH), 3055 (C-H), 1618 (C=N); ^1^H-NMR (DMSO-d_6_) δ/ppm: 3.35 (s, 3H, OCH_3_), 3.67–3.71 (m, 2H, H-5′,5’’), 3.79–3.87 (m, 2H, H-4′,3′), 3.98–4.00 (m, 1H, OH), 4.71–4.74 (m, 2H, OH, H-2′), 4.83–4.85 (m, 1H, OH), 5.82 (d, 1H, *J* = 9.6 Hz, H-1′), 7.39 (d, 2H, *J* = 8.5 Hz, Ar-H), 7.81 (d, 2H, *J* = 8.5 Hz, Ar-H), 8.55 (s, 1H, N=CH). Analysis calcd. for C_15_H_17_N_3_O_5_S_2_ (383.44): C, 46.99; H, 4.47; N, 10.96. Found: C, 47.08; H, 4.40; N, 11.09%.

#### 3.4.3. (4-Dimethylamino-benzylidene)-(5-(β-d-Galactopyranosyl)thio-[1,3,4]thiadiazol-2-yl)-amine (**10**)

Yield: 70%; yellowish foam; IR (KBr) cm^−1^, ν: 3445–3420 (OH), 3055 (C-H), 1618 (C=N); ^1^H-NMR (DMSO-d_6_) δ/ppm: 2.48, 3.03 (2s, 6H, N(CH_3_)_2_), 3.37–3.47 (m, 2H, H-6′,6′′), 3.52–3.61 (m, 1H, H-5′), 3.90–3.99 (m, 2H, H-4′,3′), 4.30–4.32 (m, 1H, OH), 4.50–4.62 (m, 2H, OH, H^2′^), 5.26–5.31 (m, 2H, 2OH), 5.79 (d, 1H, *J* = 9.4 Hz, H-1′), 6.78 (d, 2H, *J* = 8.6 Hz, Ar-H), 7.68 (d, 2H, *J* = 8.6 Hz, Ar-H), 8.55 (s, 1H, N=CH). Analysis calcd. for C_17_H_22_N_4_O_5_S_2_ (426.10): C, 47.87; H, 5.20; N, 13.14. Found: C, 48.03; H, 5.10; N, 13.26%.

#### 3.4.4. (4-Dimethylaminobenzylidene)-(5-(β-d-xylopyranosyl)thio-[1,3,4]thiadiazol-2-yl)-amine (**11**)

Yield: 78%; m.p. 152–153 °C; IR (KBr) cm^−1^, ν: 3430–3415 (OH), 3048 (C-H), 1615 (C=N); ^1^H-NMR (DMSO-d_6_) δ/ppm: 2.48, 3.03 (2s, 6H, N(CH_3_)_2_), 3.65–3.70 (m, 2H, H-5′,5’’), 3.85–3.95 (m, 2H, H-4′,3′), 3.96–4.01 (m, 1H, OH), 4.59–4.78 (m, 2H, OH, H-2′), 4.84–4.86 (m, 1H, OH), 5.91 (d, 1H, *J* = 9.8 hz, H-1′), 6.78 (d, 2H, *J* = 8.5 Hz, Ar-H), 7.75 (d, 2H, *J* = 8,5 Hz, Ar-H), 8.55 (s, 1H, N=CH). Analysis calcd. for C_16_H_20_N_4_O_4_S_2_ (396.48): C, 48.47; H, 5.08; N, 14.13. Found: C, 48.29; H, 5.16; N, 13.98%.

### 3.5. Synthesis of (4-substituted-benzylidene)-(5-prop-2-ynylsulfanyl-[1,3,4]thiadiazol-2-yl)-amine (***12***, ***13***)

A mixture of the thiol derivative **2** or **3** (2 mmol) and anhydrous potassium carbonate in DMF (20 mL) was stirred at room temperature for 30 min, then cooled to 0 °C. Propargyl bromide (2.2 mmol) was added dropwise at 0 °C over a period of 15 min and the reaction mixture was further stirred for 6 h (TLC: ethyl acetate/pet. ether; 1/3) at room temperature. Ice-cold water was added with continues shaking then the afforded precipitated solid was filtered off, washed with cold ethanol, and dried to give the acetylenic compounds **12** or **13**, respectively.

#### 3.5.1. (4-Methoxybenzylidene)-(5-prop-2-ynylsulfanyl-[1,3,4]thiadiazol-2-yl)-amine (**12**)

Yield: 72%; m.p. 134–135 °C; IR (KBr) cm^−1^, ν: 3250 (alkyne-CH), 3070 (aromatic C-H), 2118 (alkyne-CC), 1614 (C=N); ^1^H-NMR (DMSO-d_6_) δ/ppm: 1.89 (s, 1H, CH), 3.35 (s, 3H, OCH_3_), 3.84 (s, 2H, CH_2_), 7.12 (d, 2H, *J* = 8.4 Hz, Ar-H), 7.87 (d, 2H, *J* = 8.4 Hz Ar-H), 8.11 (s, 1H, N=CH). Analysis calcd. for C_13_H_11_N_3_OS_2_ (289.38): C, 53.96; H, 3.83; N, 14.52. Found: C, 54.11; H, 3.88; N, 14.30%.

#### 3.5.2. (4-Dimethylaminobenzylidene)-(5-prop-2-ynylsulfanyl-[1,3,4]thiadiazol-2-yl)-amine (**13**)

Yield: 77%; mp 139–140 °C; IR (KBr) cm^−1^, ν: 3255 (alkyne-CH), 3065 (aromatic C-H), 2120 (alkyne-CC), 1615 (C=N); ^1^H-NMR (DMSO-d_6_) δ/ppm: 1.96 (s, 1H, CH), 2.79, 2.80 (2s, 6H, N(CH_3_)_2_), 3.60 (s, 2H, CH_2_) 6.28 (d, 2H, *J* = 8.9 Hz, Ar-H), 7.30 (d, 2H, *J* = 8.9 Hz, Ar-H), 8.10 (s, 1H, N=CH). ^13^C-NMR spectrum, δ, ppm: 22.3 (CH_2_), 40.58 (2CH_3_), 75.5 (acetylene-C), 79.7 (acetylene-C), 111.5–159.9 (6Ar-C, C=N, thiadiazole-C), 168.9 (thiadiazole-C). Analysis calcd. for C_14_H_14_N_4_S_2_ (302.42): C, 55.60; H, 4.67; N, 18.53. Found: C, 55.45; H, 4.75; N, 18.41%.

### 3.6. [5-(1-(O-acetyl-β-d-glycopyranosyl)-1H-[1,2,3]triazol-4-ylmethylsulfanyl)-[1,3,4]thiadiazol-2-yl]-(4-methoxy-benzylidene)-amine (***14***–***17***)

To a well-stirred solution of the terminal alkyne derivative **12** or **13** (2.0 mmol) in THF*l*H_2_O solvent mixture (2/1; 15 mL), the glycosyl azide, 2,3,4,6-tetra-*O*-acetyl-d-glucopyranosyl or 2,3,4-tri-*O*-acetyl-d-xylopyranosyl azide (2.0 mmol), was added. Sodium ascorbate (0.4 mmol, 0.08 g) and catalytic amount of diisopropylethylamine (DIPEA) (four drops) followed by copper sulfate pentahydrate (0.4 mmol, 0.11 g) were then added, respectively. The reaction mixture was stirred at room temperature for 10 h [(TLC; pet. ether/ethyl acetate (4:1)]. Ethyl acetate (2 × 30 mL) was then provided to the mixture and the organic layer was then speared_._ The organic layers were then collected and dried over anhydrous sodium sulfate then evaporated. Further purification by column chromatography [hexane/ethyl acetate (5:1)] gave the title triazole glycosides **14**–**17**, respectively.

#### 3.6.1. [5-(1-(2,3,4,6-Tetra-*O*-acetyl-β-d-glucopyranosyl)-1*H*-[1,2,3]triazol-4-ylmethylsulfanyl)-[1,3,4]thiadiazol-2-yl]-(4-methoxybenzylidene)amine (**14**)

Yield: 75%; m.p. 106–107 °C; IR (KBr) cm^−1^, ν: 3082 (C-H), 1752 (C=O), 1618 (C=N); ^1^H-NMR (DMSO-d_6_) δ/ppm: 2.04, 2.06, 2.10, 2.13 (4s, 12H, CH_3_CO), 3.92 (s, 3H, OCH_3_), 3.38–3.41 (m, 1H, H-5′), 4.18–4.29 (dd, 1H, *J* = 2.8, *J* = 10.8 Hz, H-6*’*), 4.31–4.32 (dd, 1H, *J* = 3.2, *J* = 11.2 Hz, H-6′′), 4.66-4.68 (m, 1H, H-4′), 4.96–5.03 (dd, 1H, *J* = 8.6, *J* = 9.8 Hz, H-2′), 5.27 (t, 1H, *J* = 8.8 Hz, H-3′), 5.44 (s, 2H, CH_2_), 5.86 (d, 1H, *J* = 9.8 Hz, H-1′), 7.02 (d, 2H, *J* = 8.8 Hz, Ar-H), 7.54 (s, 1H, triazole-H), 7.88 (d, 2H, *J* = 8.8 Hz, Ar-H), 9.92 (s, 1H, N=C*H*). ^13^C-NMR spectrum, δ, ppm: 20.1, 20.5, 20.6, 20.7 (4CH_3_), 27.5 (CH_2_), 55.6 (OCH_3_), 61.7 (C-6), 67.9 (C-4), 70.7 (C-2), 74.0 (C-3), 77.2 (C-5), 97.9 (C-1), 114.3–154.4 (Ar-C, triazole-2C, thiadiazole-C), 166.5 (C=N), 169.2, 169.3, 170.1, 170.6 (4C=O, thiadiazole-C). Analysis calcd. for C_27_H_30_N_6_O_10_S_2_ (662.69): C, 48.94; H, 4.56; N, 12.68. Found: C, 48.72; H, 4.51; N, 12.80%.

#### 3.6.2. [5-(1-(2,3,4,6-Tri-*O*-acetyl-β-d-xylopyranosyl)-1*H*-[1,2,3]triazol-4-ylmethylsulfanyl)-[1,3,4]thiadiazol-2-yl]-(4-methoxybenzylidene)amine (**15**)

Yield: 77%; m.p. 101–102 °C; IR (KBr) cm^−1^, ν: 3077 (C-H), 1750 (C=O), 1612 (C=N); ^1^H-NMR (DMSO-d_6_) δ/ppm: 1.99, 2.01, 2.03 (3s, 9H, CH_3_CO), 3.88 (s, 3H, OCH_3_), 4.05–4.08 (dd, 1H, *J* = 3.4, *J* = 11.2 Hz, H-5′′), 4.25–4.27 (dd, 1H, *J* = 11.2, *J* = 3.6 Hz, H-5′), 4.57–4.70 (dd, 1H, *J* = 6.4, *J* = 8.8 Hz, H-4′), 5.14 (t, 1H, *J* = 7.8Hz, H-2′), 5.33-5.39 (m, 3H, CH_2_, H-2′), 6.17 (d, 1H, *J* = 9.6 Hz, H-1′), 6.99 (d, 2H, *J* = 7.6 Hz, Ar-H), 7.89 (d, 2H, *J* = 7.6 Hz, Ar-H), 7.91 (s, 1H, triazole-H), 8.75 (s, 1H, N=CH). Analysis calcd. for C_24_H_26_N_6_O_8_S_2_ (590.63): C, 48.81; H, 4.44; N, 14.23. Found: C, 48.67; H, 4.36; N, 14.17%.

#### 3.6.3. (4-Dimethylaminobenzylidene)-[5-(1-(2,3,4,6-tetra-*O*-acetyl-β-d-glucopyranosyl)-1*H*-[1,2,3]triazol-4-ylmethylsulfanyl)-[1,3,4]thiadiazol-2-yl]amine (**16**)

Yield: 74%; m.p. 198–199 °C; IR (KBr) cm^−1^, ν: 3075 (C-H), 1748 (C=O), 1612 (C=N); ^1^H-NMR (DMSO-d_6_) δ/ppm: 1.85, 1.88, 2.03, 2.07 (4s, 12H, CH_3_CO), 2.99, 3.13 (2s, 6H, N(CH_3_)_2_), 3.37–3.40 (m, 1H, H-5*’*), 3.99–4.32 (m,2H, H-6*’*,6′′), 4.46–4.65 (dd, 1H, *J* = 7.7, *J* = 6.8 Hz, H-4′), 5.31–5.49 (m, 3H, CH_2_, H-2′), 5.83 (t, 1H, *J* = 8.8 Hz, H-3′), 6.15 (d, 1H, *J* = 10.2 Hz, H-1′), 7.77 (d, 2H, *J* = 8.4 Hz, Ar-H), 7.85 (d, 2H, *J* = 8.4 Hz, Ar-H), 7.94 (s, 1H, triazole-H), 8.58 (s, 1H, N=C*H*). ^13^C-NMR (DMSO-d_6_) δ/ppm: 20.1, 20.2, 20.5, 20.7 (4CH_3_), 27.5 (CH_2_), 40.1 (2CH_3_), 61.5 (C-6), 67.7 (C-4), 72.7 (C-2), 75.1 (C-3), 77.1 (C-5), 96.7 (C-1), 111.0–154.4 (Ar-C, triazole-2C, thiadiazole-C), 166.51 (C=N), 168.6, 168.7, 169.3, 169.9, 170.6 (4C=O, thiadiazole-C). Analysis calcd. for C_28_H_33_N_7_O_9_S_2_ (675.73): C, 49.77; H, 4.92; N, 14.51. Found: C, 49.55; H, 4.82; N, 14.68%.

#### 3.6.4. (4-Dimethylaminobenzylidene)-[5-(1-(2,3,4-tri-*O*-acetyl-β-d-xylopyranosyl)-1*H*-[1,2,3]triazol- 4-ylmethylsulfanyl)-[1,3,4]thiadiazol-2-yl]amine (**17**)

Yield: 69%; m.p. 201–202 °C; IR (KBr) cm^−1^, ν: 3075 (C-H), 1755 (C=O), 1615 (C=N); ^1^H-NMR (DMSO-d_6_) δ/ppm: 1.85, 2.03, 2.05 (3s, 9H, CH_3_CO), 2.95, 2.97 (2s, 6H, N(CH_3_)_2_), 3.56–3.58 (dd,1H, *J* = 3.4, *J* = 11.2 Hz, H-5′′), 3.74–3.77 (dd, 1H, *J* = 11.2, *J* = 3.4 Hz, H-5′), 4.27–4.29 (dd, 1H, *J* = 6.4, *J* = 8.8 Hz, H-4′), 5.16 (t, 1H, *J* = 7.8 Hz, H-2′), 5.38–5.41 (m, 3H, CH_2_, H-3′), 5.81 (d, 1H, *J* = 9.8 Hz, H-1′), 6.72 (d, 2H, *J* = 7.6 Hz, Ar-H), 7.69 (d, 2H, *J* = 7.6 Hz, Ar-H), 7.83 (s, 1H, triazole-H), 9.74 (s, 1H, N=CH). Analysis calcd. for C_25_H_29_N_7_O_7_S_2_ (603.67): C, 49.74; H, 4.84; N, 16.24. Found: C, 49.58; H, 4.77; N, 16.18%.

### 3.7. Synthesis of [5-(1-(β-d-glycopyranosyl)-1H-[1,2,3]triazol-4-ylmethylsulfanyl)-[1,3,4]thiadiazol-2-yl]-(4-methoxybenzylidene)amine (***18***–***21***)

A solution of the protected 1,2,3-triazole glycoside derivatives **14**–**17** (2 mmol) in saturated gaseous ammonia solution in dry methanol (25 mL) was stirred at room temperature for 15 h. The solvent was evaporated at 40–45 °C and the remaining solid material was dissolved in ethanol at 40 °C, then left to stand at room temperature for 2 h. The solvent was reduced under vacuum and the precipitate was filtered and dried to give the deacetylated glycosides **18**–**21**, respectively.

#### 3.7.1. [5-(1-(β-d-glucopyranosyl)-1*H*-[1,2,3]triazol-4-ylmethylsulfanyl)-[1,3,4]thiadiazol-2-yl]-(4-methoxybenzylidene)amine (**18**)

Yield: 76%; brownish foam; IR (KBr) cm^−1^, ν: 3430–3415 (OH), 3048 (C-H), 1615 (C=N); ^1^H-NMR (DMSO-d_6_) δ/ppm: 3.23 (s, 3H, OCH_3_), 3.69–3.86 (m, 2H, H-6′,6′′), 4.11–4.13 (m, 1H, H-5′), 4.39–4.47 (m, 2H, H-4′,3′), 4.40–4.47 (m, 1H, OH), 4.65 (s, 2H, CH_2_), 5.05–5.16 (m, 2H, OH, H-2′), 5.28–5.39 (m, 2H, 2OH), 5.90 (d, 1H, *J* = 8.2 Hz, H-1′), 6.69 (s, 1H, Ar-H), 7.12 (d, 2H, *J* = 8.8 Hz, Ar-H), 7.29 (d, 2H, *J* = 8.6 Hz, Ar-H), 8.20 (s, 1H, N=CH). ^13^C-NMR (DMSO-d_6_) δ/ppm: 22.9 (CH_2_), 61.2 (OCH_3_), 61.2 (C-6), 70.0 (C-4), 72.5 (C-2), 73.8 (C-3), 76.9 (C-5), 77.4 (C-1), 114.9–154.4 (Ar-C, triazole-2C, thiadiazole-C), 171.8, 171.9 (C=N, thiadiazole-C). Analysis calcd. for C_19_H_22_N_6_O_6_S_2_ (494.54): C, 46.15; H, 4.48; N, 16.99. Found: C, 46.09; H, 4.59; N, 17.11%.

#### 3.7.2. [5-(1-(β-d-xylopyranosyl)-1*H*-[1,2,3]triazol-4-ylmethylsulfanyl)-[1,3,4]thiadiazol-2-yl]-(4-methoxybenzylidene)amine (**19**)

Yield: 68%; m.p. 154–155 °C; IR (KBr) cm^−1^, ν: 3455–3425 (OH), 3072 (C-H), 1618 (C=N); ^1^H-NMR (DMSO-d_6_) δ/ppm: 3.38 (s, 3H, OCH_3_), 3.47–3.73 (m, 2H, H-5′,5’’), 3.81–3.85 (m, 2H, H-4′,3′), 3.88–3.94 (m, 1H, OH), 4.38 (s, 2H, CH_2_), 5.17–5.30 (m, 2H, OH, H-2′), 5.39–5.40 (m, 1H, OH), 5.91 (d, 1H, *J* = 9.2 Hz, H-1′), 7.14 (d, 2H, *J* = 8.7 Hz, Ar-H), 7.31 (s, 1H, Ar-H), 7.87 (d, 2H, *J* = 8.8 Hz, Ar-H), 8.18 (s, 1H, N=CH). ^13^C-NMR (DMSO-d_6_) δ/ppm: 22.9 (CH_2_), 56.2 (OCH_3_), 68.7 (C-4), 72.4 (C-2), 77.5 (C-3), 79.6 (C-5), 88.6 (C-1), 114.9–154.4 (Ar-C, triazole-2C, thiadiazole-C), 171.8, 172.0 (thiadiazole-C, C=N). Analysis calcd. for C_18_H_20_N_6_O_5_S_2_ (464.52): C, 46.54; H, 4.34; N, 18.09. Found: C, 46.35; H, 4.18; N, 18.21%.

#### 3.7.3. (4-Dimethylaminobenzylidene)-[5-(1-(β-d-glucopyranosyl)-1*H*-[1,2,3]triazol-4-ylmethyl sulfanyl)-[1,3,4]thiadiazol-2-yl]amine (**20**)

Yield: 79%; m.p. 121–122 °C; IR (KBr) cm^−1^, ν: 3450–3425 (OH), 3070 (C-H), 1618 (C=N); ^1^H-NMR (DMSO-d_6_) δ/ppm: 2.98, 3.03 (2s, 6H, N(CH_3_)_2_), 3.40–3.42 (m, 2H, H-6′,6′′), 3.70–3.72 (m, 1H, H-5′), 4.37 (s, 2H, CH_2_), 4.55–4.62 (m, 2H, H-4′,3′), 4.79–4.81 (m, 1H, OH), 5.11–5.22 (m, 2H, OH, H-2′), 5.33–5.34 (m, 2H, 2OH), 5.90 (d, 1H, *J* = 9.1 Hz, H-1′), 7.29 (s, 1H, Ar-H), 6.78 (d, 2H, *J* = 8.6 Hz, Ar-H), 7.68 (d, 2H, *J* = 8.6 Hz, Ar-H), 8.18 (s, 1H, N=CH). Analysis calcd. for C_20_H_25_N_7_O_5_S_2_ (507.59): C, 47.33; H, 4.96; N, 19.32. Found: C, 47.42; H, 4.82; N, 19.55%.

#### 3.7.4. (4-Dimethylamino-benzylidene)-[5-(1-(β-d-xylopyranosyl)-1*H*-[1,2,3]triazol-4-ylmethyl sulfanyl)-[1,3,4]thiadiazol-2-yl]-amine (**21**)

Yield: 75%; m.p. 161–162 °C; IR (KBr) cm^−1^, ν: 3440–3420 (OH), 3065 (C-H), 1615 (C=N); ^1^H-NMR (DMSO-d_6_) δ/ppm: 3.04, 3.17 (2s, 6H, N(CH_3_)_2_), 3.44–3.46 (m, 2H, H-5′,5’’), 3.71–3.73 (m, 2H, H-4′,3′), 3.80–3.82 (m, 1H, OH), 4.37 (s, 2H, CH_2_), 5.17–5.30 (m, 2H, OH, H-2′), 5.40–5.41 (m, 1H, OH), 5.92 (d, 1H, *J* = 9 Hz, H-1′), 6.79 (d, 2H, *J* = 7.8 Hz, Ar-H), 7.32 (s, 1H, Ar-H), 7.68 (d, 2H, *J* = 7.8 Hz, Ar-H), 8.18 (s, 1H, N=CH). Analysis calcd. for C_19_H_23_N_7_O_4_S_2_ (477.56): C, 47.79; H, 4.85; N, 20.53. Found: C, 47.58; H, 4.89; N, 20.41%.

### 3.8. Materials of the Cell Lines Assay

Cell culture of HCT-116 (human colorectal carcinoma) and MCF-7 (human breast adenocarcinoma) cell lines were purchased from the American Type Culture Collection (Rockville, MD, USA) and maintained in Dulbecco’s Modified Eagle Medium (DMEM) medium which was supplemented with 10% heat-inactivated fetal bovine serum (FBS), 100 U/mL penicillin, and 100 U/mL streptomycin. The cells were grown at 37 °C in a humidified atmosphere of 5% CO_2_.

### 3.9. MTT Cytotoxic Assay

The cytotoxic activities against HCT-116 and MCF-7 human cancer cell lines were estimated using the 3-[4,5-dimethyl-2-thiazolyl)-2,5-diphenyl-2*H*-tetrazolium bromide (MTT) assay, which is based on the reduction of the tetrazolium salt by mitochondrial dehydrogenases in viable cells [39,40,41]. Cells were dispensed in a 96 well sterile microplate (1 × 10^4^ cells/well) and incubated at 37 °C with series of different concentrations, in DMSO, of each tested compound or Doxorubicin (positive control) for 48 h in a serum free medium prior to the MTT assay. After incubation, media were carefully removed, 40 µL of MTT (2.5 mg/mL) were added to each well and then incubated for an additional 4 h. The purple formazan dye crystals were solubilized by the addition of 200 µL of DMSO. The absorbance was measured at 570 nm using a Spectra Max Paradigm Multi-Mode microplate reader. The relative cell viability was expressed as the mean percentage of viable cells compared to the untreated control cells. All experiments were conducted in triplicate and repeated on three different days. All the values were represented as mean ± SD. IC_50_s were determined by probit analysis by SPSS Incprobit analysis (IBM Corp., Armonk, NY, USA).

## 4. Conclusions

New 1,3,4-thiadiazole thioglycosides and 1,2,3-triazole glycosides based substituted arylidine systems were prepared via glycosylation and click chemistry cycloaddition, respectively. In addition to the free thiadiazolethione compounds, the target glycosides possessing the 1,2,3-triazole motif via click strategy base revealed more potent activity against HCT-116 and MCF-7 human cancer cell lines, comparable to doxorubicin, than their 1,3,4-thiadiazole analogs. These results showed the importance of the triazole core in the formed glycoside compounds which have characterized with higher cytotoxic activities.

## Figures and Tables

**Table 1:** IC_50_ of the compounds against the two cancer cell lines according to the MTT assay.

Compound Code	IC_50_ (µM) ± SD	
	HCT-116	MCF-7
**2**	12.2 ± 1.3	17.2 ± 2.1
**3**	15.6 ± 2.1	16.1 ± 1.9
**4**	20.7 ± 2.4	31.4 ± 3.8
**5**	22.5 ± 3.1	23.8 ± 2.9
**6**	19.2 ± 2.5	22.4 ± 3.1
**7**	19.7 ± 2.1	23.2 ± 3.1
**10**	14.4 ± 2.1	21.7 ± 2.3
**11**	22.2 ± 3.1	34.4 ± 4.1
**12**	17.6 ± 2.3	24.3 ± 2.7
**13**	23.5 ± 2.7	24.2 ± 3.1
**14**	15.3 ± 1.9	19.3 ± 2.1
**15**	15.9 ± 2.2	20.3 ± 2.9
**16**	21.0 ± 3.1	20.7 ± 2.5
**17**	18.2 ± 2.5	22.4 ± 2.8
**18**	18.3 ± 2.2	22.5 ± 2.5
**20**	12.1 ± 1.4	17.3 ± 1.9
**21**	19.2 ± 2.5	21.5 ± 2.8
Doxorubicin	12.3 ± 1.9	20.5 ± 2.1

## References

[1] Kolb H.C., Sharpless K.B. (2003). The growing impact of click chemistry on drug discovery. Drug Discov. Today.

[2] Bourne Y., Kolb H.C., Radie Z., Sharpless K.B., Taylor P., Marchot P. (2004). Freeze-frame inhibitor captures acetylcholinesterase in a unique conformation. Proc. Natl. Acad. Sci. USA.

[3] El-Sayed W.A., Abdel-Rahman A.A.-H. (2010). Copper-catalyzed Synthesis and Antimicrobial Activity of Disubstituted 1,2,3-Triazoles Starting from 1-Propargyluracils and Ethyl (4-azido-1,2,3-trihydroxybutyl)furan-3-carboxylate. Z. Naturforsch. B.

[4] Broggi J., Joubert N., Aucagne V., Berteina-Raboin S., Diez-Gonzalez S., Nolan S., Topalis D., Deville-Bonne D., Balzarini J., Neyts J. (2007). Alkyne-Azide Click Chemistry Mediated Carbanucleosides Synthesis. Nucleosides Nucleotides Nucleic Acids.

[5] El-Sayed W.A., El-Essawy F.A., Ali O.M., Nasr B.S., Abdalla M.M., Abdel-Rahman A.A.-H. (2010). Synthesis and antiviral Evaluation of new 2,5-Disbstituted 1,3,4-Oxadiazole Derivatives and Their Acyclic Nucleoside Analogs. Monatsh. Chem..

[6] Zhu R., Wang M., Xia Y., Qu F., Neyts J., Peng L. (2008). Arylethynyltriazole acyclonucleosides inhibit hepatitis C virus replication. Bioorg. Med. Chem. Lett..

[7] Dutta S., Ji Gupta S.A., Sen K. (2016). Silver trifluoromethanesulfonate and metallic copper mediated syntheses of 1,2,3-triazole-*O*- and triazolyl glycoconjugates: Consecutive glycosylation and cyclization under one-pot condition. Tetrahedr. Lett..

[8] Zhang S.-Y., Fu D.-J., Yue X.-X., Liu Y.-C., Song J., Sun H.-H., Liu H.-M., Zhang Y.-B. (2016). Design, Synthesis and Structure-Activity Relationships of Novel Chalcone-1,2,3-triazole-azole Derivates as Antiproliferative Agents. Molecules.

[9] Ruddarraju R.R., Murugulla A.C., Kotla R., Tirumalasetty M.C.B., Wudayagiri R., Donthabakthuni S., Maroju R., Baburao K., Parasa L.S. (2016). Design, synthesis, anticancer, antimicrobial activities and molecular docking studies of theophylline containing acetylenes and theophylline containing 1,2,3-triazoles with variant nucleoside derivatives. Eur. J. Med. Chem..

[10] Dondoni A., Marra A. (2006). *C*-Glycoside Clustering on Calix [4]arene, Adamantane, and Benzene Scaffolds through 1,2,3-Triazole Linkers. J. Org. Chem..

[11] Hota S., Kashyap S. (2006). “Click Chemistry” Inspired Synthesis of *pseudo*-Oligosaccharides and Amino Acid Glycoconjugates. J. Org. Chem..

[12] Mohamed A.M., Al-Qalawi H.R.M., El-Sayed W.A., Arafa W.A.A., Alhumaimmis M.S., Hassan A.K. (2015). Anticancer activity of newly synthesized triazolopyrimidine derivatives and their nucleoside analogs. Acta Pol. Pharm..

[13] Carvalho I., Andrade P., Campo V.L., Guedes P.M., Sesti-Costa R., Silva J.S., Schenkman S., Dedola S., Hill L., Rejzek M. (2010). ‘Click chemistry’ synthesis of a library of 1,2,3-triazole-substituted galactose derivatives and their evaluation against Trypanosoma cruziand its cell surface trans-sialidase. Bioorg. Med. Chem..

[14] Agalave S.G., Maujan S.R., Pore V.S. (2011). Click chemistry: 1,2,3-triazoles as pharmacophores. Chem. Asian J..

[15] Li Y., Geng J., Liu Y., Yu S., Zhao G. (2013). Thiadiazole-A Promising Structure in Medicinal Chemistry. Chem. Med. Chem..

[16] Hu Y., Li C.Y., Wang X.M., Yang Y.H., Zhu H.L. (2014). 1,3,4-Thiadiazole: Synthesis, Reactions, and Applications in Medicinal, Agricultural, and Materials Chemistry. Chem. Rev..

[17] Matysiak J. (2015). Biological and Pharmacological Activities of 1,3,4-Thiadiazole Based Compounds. Mini-Rev. Med. Chem..

[18] Haider S., Alam M.S., Hamid H. (2015). 1,3,4-Thiadiazoles: A Potent Multi Targeted Pharmacological Scaffold. Eur. J. Med. Chem..

[19] Aliabadi A. (2016). 1,3,4-Thiadiazole Based Anticancer Agents. Anti-Cancer Agents. Med. Chem..

[20] El-Sayed W.A., Metwally M.A., Nada D.S. (2013). Abdel-Rahman A.A.-H. Synthesis and Antimicrobial Activity of New Substituted 5-(Pyridine-3-yl)-1,3,4-thiadiazoles and Their Sugar Derivatives. J. Heterocycl. Chem..

[21] Zhang K., Wang P., Xuan L.N., Fu X.Y., Jing F., Li S., Liu Y.M., Chen B.Q. (2014). Synthesis and Antitumor Activities of Novel Hybrid Molecules Containing 1,3,4-Oxadiazole and 1,3,4-Thiadiazole Bearing Schiff Base Moiety. Bioorg. Med. Chem. Lett..

[22] Yadagiri B., Gurrala S., Bantu R., Nagarapu L., Polepalli S., Srujana G., Jain N. (2015). Synthesis and Evaluation of Benzosuberone Embedded with 1,3,4-Oxadiazole, 1,3,4-Thiadiazole and 1,2,4-Triazole Moieties as New Potential Anti Proliferative Agents. Bioorg. Med. Chem. Lett..

[23] Li Y.J., Qin Y.J., Makawana J.A., Wang Y.T., Zhang Y.Q., Zhang Y.L., Yang M.R., Jiang A.Q., Zhu H.L. (2014). Synthesis, Biological Evaluation and Molecular Modeling of 1,3,4-Thiadiazol-2-amide Derivatives as Novel Antitubulin Agents. Bioorg. Med. Chem..

[24] Guan P., Wang L., Hou X., Wan Y., Xu W., Tang W., Fang H. (2014). Improved Antiproliferative Activity of 1,3,4-Thiadiazole-Containing Histone Deacetylase (HDAC) Inhibitors by Introduction of the Heteroaromatic Surface Recognition Motif. Bioorg. Med. Chem..

[25] Hosseinzadeh L., Khorand A., Aliabadi A. (2013). Discovery of 2-Phenyl-*N*-(5-(trifluoromethyl)-1,3,4-thiadiazol-2-yl)acetamide Derivatives as Apoptosis Inducers via the Caspase Pathway with Potential Anticancer Activity. Arch. Pharm. Chem. Life Sci..

[26] Jordheim L.P., Durantel D., Zoulim F., Dumontet C. (2013). Advances in the development of nucleoside and nucleotide analogues for cancer and viral diseases. Nat. Rev. Drug. Disc..

[27] Zi C.-T., Li G.-T., Li Y., Zhou J., Ding Z.-T., Jiang Z.-H., Hu J.-M. (2015). Synthesis and Anticancer Activity of 4*β*-Triazole-podophyllotoxin Glycosides. Nat. Prod. Bioprospect..

[28] Flefel E.M., El-Sayed W.A., El-Sofany W.I., Mohamed A.M., Awad H.M. (2017). Synthesis and Anticancer Activity of New 1-Thia-4-azaspiro[4,5]decane, Their Derived Thiazolopyrimidine and 1,3,4-Thiadiazole Thioglycosides. Molecules.

[29] El-Sayed W.A., Fathi N.M., Gad W.A., El-Ashry E.S.H. (2008). Synthesis, and Antiviral Evaluation Of Some 5-*N*-Arylaminomethyl-2-glycosylsulphanyl-1,3,4-oxadiazoles and their analogues against Hepatitis A and Herpes simplex viruses. J. Carbohydr. Chem..

[30] El-Sayed W.A., Abdel Megeid R.E., Abbas H.S. (2011). Synthesis and Antimicrobial Activity of New 1-[(tetrazol-5-yl)methyl]indole Derivatives, their 1,2,4-Triazole Thioglycosides and Acyclic Analogs. Arch. Pharm. Res..

[31] Flefel E.M., Tantawy W.A., El-Sayed W.A., Sayed H.H., Fathy N.M. (2013). Synthesis and Anticancer Activity of New Substituted Pyrazoles and Their Derived 1,2,4-Triazoles and Their Sugar Derivatives. J. Heterocycl. Chem..

[32] El-Sayed W.A., El-Sofany W.I., Hussein H.A., Fathi N.M. (2017). Synthesis and Anticancer Activity of New [(Indolyl)pyrazolyl] -1,3,4-oxadiazole thioglycosides and Acyclic Nucleoside Analogs. Nucleosides Nucleotides Nucleic Acids.

[33] El-Sayed W.A., Rashad A.E., Awad S.M., Ali M.M. (2009). Synthesis and in vitro Anti-tumor Activty of some New Substituted thiopyrimidines through Radical Balance Regulation. Nucleosides Nucleotides Nucleic Acids.

[34] Kassem A.F., Abbas E.M.H., El-Kady D.S., Awad H.M., El-Sayed W.A. (2019). Design, Synthesis and Anticancer Activity of New Thiazole-Tetrazole or Triazole Hybrid Glycosides Targeting CDK-2 via Structure-Based Virtual Screening. Mini-Rev. Med. Chem..

[35] Mansour A.K., Ibrahim Y.A., Khalil N.S.A.M. (1999). Selective synthesis and structure of 6-arylvinyl-2- and 4-glucosyl-1,2,4-triazines of expected interesting biological activity. Nucleosides Nucleotides Nucleic Acids.

[36] El-Sayed W.A., Khalaf H.S., Mohamed S.F., Hssien H.A., Kutkat O.M., Amr A.E.-G. (2017). Synthesis and Antiviral Activity of 1,2,3-Triazole Glycosides Based Substituted Pyridine Via Click Cycloaddition. Russ. J. Gen. Chem..

[37] Saman E., Claeyssens M., Kersters-Hilderson H., De Bruyne C.K. (1973). Azido compounds as potential affinity labels for glycosidases. Carbohydr. Res..

[38] Adesoye O.G., Mills I.N., Temelkoff D.P., Jackson J.A., Norris P. (2012). Synthesis of a d-Glucopyranosyl Azide: Spectroscopic Evidence for Stereochemical Inversion in the S_N_2 Reaction. J. Chem. Educ..

[39] Hassan A.S., Mady M.F., Awad H.M., Hafez T.S. (2017). Synthesis and antitumor activity of some new pyrazolo [1, 5-a] pyrimidines”. Chin. Chem. Lett/.

[40] Emam A.N., Loutfy S.A., Mostafa A.A., Awad H.M., Mohamed M.B. (2017). Cyto-toxicity, biocompatibility and cellular response of carbon dots–plasmonic based nano-hybrids for bioimaging”. RSC Adv..

[41] El-Sayed W.A., Khalaf H.S., Osman D.A.A., Abbas H.S., Ali M.M. (2017). Synthesis and Anticancer Activity of New Pyrazolyl and Oxadiazolyl Glycosides Based on Theinopyrimidine Nucleus and Their Acyclic Analogs. Acta Pol. Pharm Drug Res..

